# How Does Health Status Affect Marginal Utility of Consumption? Evidence from China

**DOI:** 10.3390/ijerph17072234

**Published:** 2020-03-26

**Authors:** Xiaoyu Wang, Chunan Wang

**Affiliations:** 1 National School of Development, Peking University, Beijing 100871, China; chunzhifuxiao@163.com; 2 School of Economics and Management, Beihang University, Beijing 100191, China

**Keywords:** health status, marginal utility of consumption, CHARLS, China

## Abstract

This paper investigates how the deteriorating health status of an individual affects the marginal utility of non-medical consumption in China. By using 2011, 2013 and 2015 China Health and Retirement Longitudinal Study (CHARLS) data, we find that when the number of chronic diseases increases one standard deviation, the marginal utility of consumption will increase by 16.0% and 20.0% for samples of the middle-aged and elderly individuals over 50 and 65 years of age, respectively. This result is to some extent contrary to the findings from the US. Different economic development stages, intergenerational norms and bequest motives may be reasons for these contrasting patterns between China and the US.

## 1. Introduction

One of the most important factors determining economic development is consumption, thus, studies on the influence of an individual’s health status on consumption are required. Numerous studies have investigated the effect of deteriorating health on household well-being [1,2,3,4], both in high-income countries [5,6] and low- and middle-income countries [7,8,9,10,11,12]. In particular, in low- and middle-income countries, the source of a large share of household medical expenses is found to be restricted consumption, and thus the deteriorating health status may significantly reduce household consumption [13,14,15,16,17,18]. Such a pattern was found in, among others, China [12], Iran [19], Jamaica [20], Malawi [21], and Tanzania [22]. In fact, according to the economic theory, individuals’ decisions on how much to consume depends crucially on the marginal utility of consumption. If an individual’s marginal utility of consumption becomes higher, she/he may have a greater incentive to consume. Accordingly, focusing on the relationship between the health status and the marginal utility of consumption may be an appropriate approach in order to understand how health affects consumption.

A more urgent motivation to study the relationship between health and marginal utility of consumption is that such relationship plays an important role in the understandings of, among others, health care contracts and saving behavior (see Supplementary Materials file). The assumption in many applied studies is that the shape of the utility function is independent of health which in fact results in biased estimations and suboptimal mechanism designs [23,24,25,26,27]. By using the Health and Retirement Study (HRS), it was found that in the US, the marginal utility of non-medical consumption declines as health deteriorates [23]. Few studies discuss the relationship between health and the marginal utility of consumption in low- and middle-income countries, such as China. In fact, such a relationship could be important for countries like China. Especially against the background of rapid population ageing, the design of health insurance, the estimation of healthcare demand and saving behavior, which may be affected significantly by such relationship, are all vitally important determinants for social welfare and economic development.

Therefore, by using an intertemporal choice model and 2011, 2013 and 2015 China Health and Retirement Longitudinal Study (CHARLS) data, this paper aims to investigate how changes in health status may affect the marginal utility of non-medical consumption. There exist a series of methods in the literature to estimate the relationship between health status and the shape of the utility function [28,29,30,31,32,33]. In order to compare with the results found in the US [23], we use a similar method, that is, the variation in changes in subjective well-being in response to health shocks across individuals from different income levels. By estimating a linear probability model, we find that when the number of chronic diseases increases one standard deviation, the marginal utility of consumption will increase by 16.0% and 20.0% for samples of the middle-aged and elderly individuals over 50 and 65 years of age, respectively. This result is to some extent in contrast to the results found in the US [23]. Different economic development stages, intergenerational norms and bequest motives may be reasons for these contrasting patterns between China and the US.

We then extend the baseline regression. For example, we distinguish between symptomatic and asymptomatic chronic diseases; we consider two sub-samples, individuals who received education and never received education; and we focus on individuals’ physical condition. In particular, when the physical function of an individual gradually weakens, the marginal utility of consumption decreases. We also conduct a series of robustness analyses and find that most of our results are robust to the inclusion of psychiatric disease and hukou (i.e., a system of household registration in China), various consumption and utility proxies, and the use of logit and probit models.

The rest of the paper is organized as follows: Section 2 introduces the theoretical framework. Section 3 conducts the empirical analysis. Finally, Section 4 concludes and discusses policy implications.

## 2. Theoretical Framework

The theoretical framework is based on an intertemporal choice model, which has been used in the relevant literature [23,34]. In this two-period consumer choice model, an individual maximizes the expected total utility. Denoting an individual’s health status by *S*, individuals are always healthy in the first period (*S* = 0), while they become sick (*S* = 1) with probability 0 < *p* < 1 in the second period. Individuals’ lifetime utility is the sum of the utility from the non-medical consumption in the first period and the expected utility in the second period, that is:

(1) $$U\left(C_{1},C_{2},S\right)=u\left(C_{1}\right)+\frac{1}{1+\delta }E_{1}\left[u\left(C_{2},S\right)\right]$$

in which $C_{1}$ and $C_{2}$ are non-medical consumptions in the first and second periods, respectively, and *δ* is the discount rate. Based on the concern of analytical tractability of the model and the stylized facts in China (see the Supplementary Materials file), we assume that all individuals are fully insured in terms of medical cost, or equivalently, individuals do not have out-of-pocket medical expenditure.

The first- and second-period utility functions in Equation (1) are in the form of constant relative risk aversion (CRRA), with:

(2) $$u\left(C_{1}\right)=\frac{1}{1-\gamma }C_{1}^{1-\gamma }$$

(3) $$u\left(C_{2},S\right)=\varphi_{0}S+\left(1+\varphi_{1}S\right)\frac{1}{1-\gamma }C_{2}^{1-\gamma }$$

in which *γ* > 0 is the coefficient of relative risk aversion; and $\varphi_{0}$ and $\varphi_{1}$ are parameters. Health status affects the second-period utility either through the non-medical consumption ($\varphi_{1}$) or through the direct benefit from medical services, given sickness ($\varphi_{0}$). By using Equation (3), the expected utility in the second period is:

(4) $$E_{1}\left[u\left(C_{2}\right)\right]=\left(1-p\right)\frac{1}{1-\gamma }C_{2}^{1-\gamma }+p\left[\varphi_{0}+\left(1+\varphi_{1}\right)\frac{1}{1-\gamma }C_{2}^{1-\gamma }\right]\;=p\varphi_{0}+\left(1+p\varphi_{1}\right)\frac{1}{1-\gamma }C_{2}^{1-\gamma }$$

Letting *r* denote the real interest rate and *Y* denote the income, the intertemporal budget constraint is:

(5) $$Y=C_{1}+\frac{1}{1+r}C_{2}$$

which gives:

(6) $$C_{1}=Y-\frac{1}{1+r}C_{2}$$

Plugging $C_{1}$ in Equation (6) into Equation (2) and combining Equations (1), (2) and (4), an individual’s expected total utility is:

(7) $$\begin{array}{ll} E_{1}\left[U\left(C_{1},C_{2},S\right)\right] & =E_{1}\left[U\left(C_{2}\right)\right]\\ & =\frac{1}{1-\gamma }{\left(Y-\frac{1}{1+r}C_{2}\right)}^{1-\gamma }+\frac{1}{1+\delta }\left[p\varphi_{0}+\left(1+p\varphi_{1}\right)\frac{1}{1-\gamma }C_{2}^{1-\gamma }\right] \end{array}$$

An individual maximizes the expected total utility in Equation (7) by choosing $C_{2}$. Thus, the optimal second-period non-medical consumption $C_{2}^{*}$ is:

(8) $$C_{2}^{*}=\frac{{\left[\frac{\left(1+r\right)\left(1+p\varphi_{1}\right)}{1+\delta }\right]}^{\frac{1}{\gamma }}}{1+\frac{1}{1+r}{\left[\frac{\left(1+r\right)\left(1+p\varphi_{1}\right)}{1+\delta }\right]}^{\frac{1}{\gamma }}}Y\equiv cY$$

in which *c* is the parameter determining the relationship between $C_{2}^{*}$ and *Y*. As shown in Equation (8), the optimal second-period non-medical consumption $C_{2}^{*}$ is a linear function of income *Y*.

Plugging $C_{2}^{*}$ into the second-period utility function in Equation (3), the second-period indirect utility function will be:

(9) $$v\left(Y,S\right)=\left(\frac{c^{1-\gamma }}{1-\gamma }\right)\varphi_{1}SY^{1-\gamma }+\varphi_{0}S+\left(\frac{c^{1-\gamma }}{1-\gamma }\right)Y^{1-\gamma }$$

Rewriting v(Y,S) in Equation (9) gives the following non-linear regression:

(10) $$v=\beta_{1}S\times Y^{\beta_{2}}+\beta_{3}S+\beta_{4}Y^{\beta_{2}}+\eta$$

in which $\beta_{1}$, $\beta_{2}$, $\beta_{3}$, and $\beta_{4}$ are relevant parameters, with:

(11) $$\beta_{1}\equiv \left(\frac{c^{1-\gamma }}{1-\gamma }\right)\varphi_{1}$$

(12) $$\beta_{2}\equiv 1-\gamma$$

(13) $$\beta_{3}\equiv \varphi_{0}$$

(14) $$\beta_{4}\equiv \frac{c^{1-\gamma }}{1-\gamma }$$

## 3. Empirical Analysis

### 3.1. Empirical Specification

The data sources of this paper are the CHARLS 2011, 2013 and 2015 studies, which record the information of individuals and households within a period of four years only. Due to this time limitation, the variation of health status for each individual is not significant. Thus, considering the characteristics of the data, we will not control for the individual fixed effect in the empirical analysis.

The non-linear regression in Equation (10) can be written as:

(15) $$UtilityProxy_{it}=g\left(\beta_{1}S_{it}\times Y_{it}^{\beta_{2}}+\beta_{3}S_{it}+\beta_{4}Y_{it}^{\beta_{2}}+X_{it}\Gamma +\alpha_{it}\right)$$

in which g(·) is a monotonically increasing mapping from latent von Neumann-Morgenstern utility to the utility proxy; $X_{it}$ is a vector of control variables; and Γ is a vector of parameters.

As the dependent variable in the baseline regression is a binary choice (0 and 1), we will mainly use the linear probability model. Assume that the coefficient of relative risk aversion is 1, that is, γ→1 and $\beta_{2}\to 0$ [35,36]. Hence, according to L’Hôpital’s rule, the CRRA utility function can be written in the form of a logarithm. Assume that g(·) is a linear function [23]. We will then estimate the following linear probability model:

(16) $$E\left(UtilityProxy_{it}|S_{it},Y_{it},X_{it}\right)=\beta_{1}S_{it}\times \ln Y_{it}+\beta_{3}S_{it}+\beta_{4}\ln Y_{it}+X_{it}\Gamma$$

Because of the significant measurement errors of consumption data existing in CHARLS, we use the income $Y_{it}$ to be a proxy of non-medical consumption [23], which will be discussed in detail later. Taking the partial derivative of the expectation of utility in Equation (16) with respect to the logarithm of income, we can see that when the non-medical consumption changes 1%, the expectation of utility will change $\beta_{1}S_{it}+\beta_{4}$. Consequently, the effect of health status on the marginal utility of consumption can be shown by $\beta_{1}$.

If an individual is healthy ($S_{it}=0$), the marginal utility of consumption is $\beta_{4}$. If an individual is sick ($S_{it}=1$), the marginal utility of consumption is $\beta_{1}+\beta_{4}$. Therefore, when an individual’s health status $S_{it}$ changes from 0 to 1, the percentage change in the marginal utility of consumption is $\beta_{1}/\beta_{4}$. If we further consider the standard deviation of health status $\sigma_{health}$, when the health status changes one standard deviation, the percentage change in the marginal utility of consumption is $\sigma_{health}\beta_{1}/\beta_{4}$.

In order to guarantee the robustness of results, we also identify the effect of health status on the marginal utility of consumption by exploiting logit and probit models, when we assume that the error term $\alpha_{it}$ in Equation (15) follows a logistic distribution and a standard normal distribution, respectively. Note that in these two models, we calculate and report the average marginal effect.

### 3.2. Data

The data sources are the 2011, 2013 and 2015 China Health and Retirement Longitudinal Study (CHARLS). CHARLS is a nationwide Chinese household survey, conducted by the National School of Development of Peking University, that aims to survey Chinese citizens over 45 years of age, with a priority on health and retirement. One of the most significant advantages of using CHARLS is that its questionnaire design refers to international experience; for example, Health and Retirement Study (HRS) in the US, Survey of Health, Aging and Retirement in Europe (SHARE) in Europe and English Longitudinal Study of Aging (ELSA) in the UK. Hence, studies using CHARLS are easier to compare within the literature. CHARLS 2011, 2013 and 2015 received ethical approval from the institutional review board of Peking University Health Science Center. In this study, we use a sample of the middle-aged and elderly individuals over 50 years of age, which involves 18,100 individuals and 37,856 observations. In order to compare this with the existing study in the US [23], we use two sub-samples; middle-aged and elderly individuals over 50 and 65 years of age (denoted by “Sample 50” and “Sample 65”, respectively). The variables include proxies of utility, health, consumption, and individual and household characteristics.

#### 3.2.1. Utility

In the baseline regression, we use subjective well-being as the proxy of individual utility. Specifically, we use the question “I was happy during the last week” and construct the variable “HAPPY”. HAPPY is 1 if an individual chooses “most or all of the time (5–7 days)”, and 0 otherwise. Note that although this variable is controversial in the literature, the major concern for using happiness to measure utility is the precision instead of the unbiasedness. [23,37] In the robustness analysis, we use Center for Epidemiologic Studies Depression scales (CESD8 and CESD4) as utility proxies. The construction of CESD8 includes the following questions: during the last week “I felt depressed”, “I felt everything I did was an effort”, “I felt hopeful about the future”, “I felt fearful”, “My sleep was restless”, “I was happy”, “I felt lonely”, and “I could not get going”. CESD4 is a subset of CESD8, including the following questions: during the last week “I felt hopeful about the future”, “I felt fearful”, “I was happy”, and “I felt lonely”. The greater CESD8/CESD4 is, the better mental state (higher utility) will be. Note also that even though CESD variables are measures of health status, they can also be regarded as a generalized measure of subjective well-being and thus also be used as proxies of utility [23].

#### 3.2.2. Health

We do not use subjective proxies for health, such as a self-assessment of health status, because subjective variables for both dependent and explanatory variables may involve correlated measurement errors. Instead, in the baseline regression, we use the number of chronic diseases, that is, the variable “CHRONIC DISEASE”, to measure an individual’s health status. We consider 13 types of chronic diseases, including hypertension, dyslipidemia, diabetes, cancer, lung disease, liver disease, heart attack, stroke, kidney disease, stomach disease, memory-related disease, arthritis, and asthma. As psychiatric disease may affect an individual’s cognition, we do not consider it in the number of chronic diseases in the baseline regression. In fact, our result is robust to the inclusion of psychiatric disease, which will be shown in the robustness analysis.

We further distinguish between symptomatic and asymptomatic chronic diseases, as these two types of chronic diseases may have differentiated effects on the marginal utility of consumption. Symptomatic diseases refer to those that an individual may clearly identify, which include lung disease, stroke, arthritis, and asthma. However, for asymptomatic diseases, the relevant symptom may not be easy to identify. Asymptomatic diseases include hypertension, heart attack, diabetes, memory-related disease, dyslipidemia, liver disease, kidney disease, and stomach disease. As it is controversial whether cancer is a kind of symptomatic or asymptomatic disease, we run two regressions considering both cases. In addition, we also use each chronic disease to be the proxy of health status.

In the extension analysis, we also focus on physical condition, one aspect of health status, in order to study how the marginal utility of consumption will change if an individual loses physical function to some extent. Specifically, we use Activities of Daily Living (ADL), Instrumental Activities of Daily Living (IADL) and Other Functional Limitation (OFL) as proxies of health status. The greater ADL/IADL/OFL is, the worse health status will be. Specifically, ADL includes “dress”, “bathe”, “eat”, “get in and out of bed”, “use the toilet”, and “control urination and defecation”. IADL includes “prepare meals”, “shop for groceries” and “take medications”. It is worth noting that the questionnaires of CHARLS 2011 and 2015 include some other items of IADL. Nevertheless, for the sake of consistency, we only use the three indicators mentioned above. Finally, OFL includes “run about 1 km”, “walk 1 km”, “walk 100 m”, “stand”, “climb several flights of stairs”, “stoop/kneel/crouch”, “extend arms above shoulder level”, “lift or carry weights over 10 jin (i.e., a Chinese weight measure)”, and “pick up a small coin from a table”.

#### 3.2.3. Consumption

There exists households’ consumption information in CHARLS. However, when individuals respond to the questionnaire, they must recall a large number of categories of household consumption, which implies that the consumption data in CHARLS is far less accurate than the income data. As a result, even though it is ideal to use directly the consumption data and the correlation between consumption and income may probably be not perfectly linear, because of more severe measurement errors in consumption data, following the relevant study in the US, we use the household income, that is, the variable “HOUSEHOLD INCOME”, as the proxy of consumption [23]. The sources of household income include non-labor income (pension, transfer from family members, friends and other non-relatives, and public transfer), labor income and 5% of the value of financial assets. More details about income sources are in the Supplementary Materials file. Note that the relevant study in the US uses the average across different waves of annual household non-labor income plus 5% of the value of financial assets as the proxy [23]. Nonetheless, considering the characteristics of Chinese households, we choose to use the annual household income. In fact, because the social security system in China has not been developed sufficiently, many middle-aged and elderly individuals still work after retirement, obtaining labor income in order to support a significant share of their consumption. For this reason, we include labor income into the variable “HOUSEHOLD INCOME” and do not take the average across different waves of annual household income. As middle-aged and elderly individuals may start to consume their financial assets, we include 5% of financial asset value into the annual household income. In addition, inflation is treated in the construction of the variable. Finally, considering outliers, we exclude the first and last 1% of annual household income.

For the sake of robustness, we also use the annual household non-labor income (“HOUSEHOLD NL INCOME”), the average across three waves of annual household income (“AVE HOUSEHOLD INCOME”), the average across three waves of annual household non-labor income (“AVE HOUSEHOLD NL INCOME”), education (“EDUCATION”), and residential location (“RESIDENTIAL LOCATION”) as proxies of consumption variable, in which EDUCATION equals 1 if an individual received education, and 0 otherwise; and RESIDENTIAL LOCATION equals 1 if an individual lives in urban areas, and 0 otherwise.

#### 3.2.4. Individual and Household Characteristics

In order to capture individual characteristics, we use gender, age, age squared, and marriage situation in the baseline regression, as well as hukou in the robustness analysis. Specifically, for the variable “FEMALE”, it equals 1 if an individual is a female, and 0 otherwise. For the variable “MARRIED”, it equals 1 if an individual is married, and 0 otherwise. In addition, for the variable “HUKOU”, it equals 1 if an individual’s hukou belongs to the non-agriculture hukou, and 0 otherwise. Finally, we use the variable “HOUSEHOLD SIZE”, which is the number of family members who live together, to capture household characteristics.

#### 3.2.5. Summary Statistics

The summary statistics of variables are shown in Table 1. Both Samples 50 and 65 consist of around 50% female participants. Moreover, around 40% of individuals feel happy most or all of the time. The average number of chronic diseases and ADL/IADL/OFL of Sample 50 are less than those of Sample 65, respectively, while the annual household income of Sample 50 is greater than that of Sample 65, which show that both health status and income of individuals deteriorate with age. Individuals are better educated in Sample 50 than Sample 65, while around 38% of individuals in both Samples 50 and 65 live in urban areas. Moreover, the household size of Sample 50 is 3.417, which is larger than that of Sample 65. Finally, for Sample 50, the average age of individuals is 62.421 and 85.7% of individuals are married, while for Sample 65, the average age is 71.828 and only 74.2% of individuals are married. To summarize, the summary statistics of variables shown in Table 1 are consistent with the intuition for the difference between Samples 50 and 65.

### 3.3. Some Discussions on Identification

Due to the limited number of waves in CHARLS and characteristics of Chinese households mentioned above, the design of our study deviates to some extent from the relevant study in the US [23]. Therefore, there is necessity to compare the design of studies in the US and China, and discuss more the identification.

In the relevant study in the US, the proxy of consumption is the average across different waves of annual household non-labor income plus 5% of the value of financial assets, that is, the consumption across different waves is almost the same for a household, which implies that the variation of health status does not have a first-order effect on consumption. Then, by using a within-subject identification strategy, the change of marginal utility of consumption can be clearly attributed to the change health status of an individual.

However, due to the short time span of the currently available CHARLS data, on the one hand, the variation of health status of an individual is not significant, and on the other hand, the sample size will decrease significantly and we will lose much information if we stick to the within-subject strategy. Accordingly, pooling observations in all three waves together and conducting a cross-sectional study, that is, a between-subject identification strategy, might be more appropriate for China. Due also to the fact that we include labor income into the proxy of consumption and do not take the average across different waves of annual household income as we discussed, individuals’ health status does have a first-order effect on consumption. Undoubtedly, such study design will lead to a confounding effect. Specifically, as consumption varies with deteriorating health, it might be difficult to distinguish between the direct effect of health on the marginal utility of consumption, which is perfectly estimated by the relevant study in the US, and the indirect effect of health via the variation in the amount of consumption. Fortunately, we conduct ANOVA tests and find that, for our samples, when the health status changes marginally, the amount of consumption on average does not vary significantly. As a result, the coefficients estimated may still illustrate the direct effect on the marginal utility of consumption of health. In a word, even though there exists a deviation from the relevant study in the US, our identification strategy can still show the desired effect. In fact, we also follow exactly the identification strategy of the relevant study in the US for robustness. The results of robustness analysis will be discussed later.

### 3.4. Results and Discussion

#### 3.4.1. Baseline Regression

In the baseline regression, we estimate a linear probability model, in which the dependent variable is the binary choice “I was happy during the last week”, and explanatory variables include the number of chronic diseases, annual household income, the interaction between the number of chronic diseases and annual household income, gender, age, age squared, marriage situation, and household size.

The first column of Table 2 gives the estimation result of baseline regression, which reports the robust standard errors. The second column reports the cluster-robust standard errors. The third column controls the provincial fixed effect, reporting the robust standard errors. We find that the estimation results in three columns are similar. Accordingly, we use the estimation results of baseline regression, that is, the first column of Table 2, as an example to analyze.

The parameters of interest are $\beta_{1}$ and $\beta_{4}$. According to the estimation results of baseline regression in Table 2, the coefficients of the interaction term ($\beta_{1}$) of Samples 50 and 60 are 0.003 and 0.004, respectively; statistically significant at 1% and 5% significance levels, respectively. These results show that the deterioration of health status can raise the marginal utility of non-medical consumption. The coefficients of logarithm of annual household income ($\beta_{4}$) of Samples 50 and 60 are around 0.027 and 0.030, respectively; both statistically significant at 1% significance level, implying that when consumption increases by 10%, the probability of reporting HAPPY most or all of the time in Sample 50 will increase by 0.27%, while that probability in Sample 65 will increase by 0.30%. Consequently, the increase of consumption can raise individual utility, and the positive effect of consumption on utility is more significant in the elderly sample (Sample 65). Furthermore, the coefficients of chronic disease ($\beta_{3}$) of Samples 50 and 60 are around −0.072 and −0.080, respectively; both statistically significant at 1% significance level, showing that the deterioration of health status can significantly decrease individual utility, and the negative effect of deteriorating health on utility is more significant in the elderly sample (Sample 65).

The coefficients of females in Samples 50 and 65 are −0.029 and −0.026; both statistically significant at a 1% significance level, showing that the individual utility will decrease if the individual is female. In fact, middle-aged and elderly females in China usually undertake more housework than males, implying lower individual utility. The coefficients of age and age squared are only statistically significant in Sample 65. Specifically, for this sample, the coefficients of age and age squared are 0.041 and −0.000, respectively; both statistically significant at 5% significance level, implying that the increase of age will raise utility but at a decreasing rate. For Samples 50 and 65, the coefficients of marriage are 0.065 and 0.039, respectively; both statistically significant at 1% significance level. These results show that a married individual has a higher degree of happiness than a single individual. Finally, the coefficients of household size show that the greater the number of family members living together, the lower utility an individual has. The reason for the coefficients of household size may also be related to the housework.

As we have discussed in the empirical specification, when the number of chronic diseases increases one unit, the percentage change of the marginal utility of consumption is $\beta_{1}/\beta_{4}$; when the number of chronic diseases increases one standard deviation, the percentage change of the marginal utility of consumption is $\sigma_{health}\beta_{1}/\beta_{4}$, in which $\sigma_{health}$ is the standard deviation of the number of chronic diseases. According to the estimation results of baseline regression, Table 3 presents the calculations of coefficient ratios, using the method of bootstrap resampling.

According to Table 3, for Sample 50, the ratio $\beta_{1}/\beta_{4}$, statistically significant at 5% significance level, shows that when the number of chronic diseases increases one unit, the marginal utility of consumption will increase by 11.7%, in which the bootstrapped standard error is 5.1% and the bootstrapped confidence interval is between 1.6% and 21.7%. For Sample 65, the ratio $\beta_{1}/\beta_{4}$, statistically significant at 10% significance level, shows that when the number of chronic diseases increases one unit, the marginal utility of consumption will increase by 14.1%, in which the bootstrapped standard error is 8.6% and the bootstrapped confidence interval is between -2.7% and 30.9%. In the meantime, $\sigma_{health}\beta_{1}/\beta_{4}$ implies that when the number of chronic diseases increases one standard deviation, the marginal utility of consumption will increase by 16.0% for Sample 50 and 20.0% for Sample 65. We also calculate the values of marginal utility of consumption under different numbers of chronic diseases. For Sample 50, when the number of chronic diseases increases from 0 to 5, the marginal utility of consumption will be 0.027, 0.030, 0.033, 0.036, 0.039, and 0.042, respectively. Similarly, for Sample 65, the marginal utility of consumption will be 0.030, 0.034, 0.038, 0.042, 0.046, and 0.050, respectively. These results show that when the health status deteriorates, the marginal utility of non-medical consumption will increase.

The results in Table 2 and Table 3 are to some extent contrary to the corresponding findings from the US [23]. Therefore, it is interesting to see the possible reasons for the differences between China and the US. First, such results in China may probably not come from the decrease in consumption. According to economic theory, the marginal utility of consumption decreases with consumption. In our samples, the amount of consumption of individuals decrease with deteriorating health. Thus, it might be possible that when the health status of the middle-aged and elderly individuals in China becomes worse, their consumption will decrease and then their marginal utility of consumption will increase. In fact, we conduct ANOVA tests and find that, for groups of individuals with adjacent numbers of chronic diseases, the average amounts of consumption are not statistically significant at 5% significance level. In other words, for our samples, when the number of chronic diseases increases one unit, the amount of consumption on average does not decrease significantly. Consequently, the channel through decreasing consumption might not explain well our results shown in Table 2 and Table 3.

There exist various reasons for the differences between China and the US. Economic development stages may be a possible reason. In high-income countries, such as the US, if an individual’s health deteriorates, the middle-aged and elderly may no longer be able to continue their more expensive consumption (e.g., in tourism activities), which is a complementary product for good health. Thus, if the marginal utility of the consumption of substitute products for good health is dominated by complementary products, the marginal utility of consumption may decline with deteriorating health. At present, most middle-aged and elderly individuals in the low- and middle-income countries, such as China, were born before the 1960s and may have experienced poverty in their youth: this may consequently lead to more frugal consumption in their later life. Nonetheless, after a deterioration in health, they may pay more attention to, for example, eating more healthily or making their living conditions more suitable to manage deteriorating health conditions. In this way, when their health does deteriorate, the marginal utility of the consumption of substitute products for good health may dominate the marginal utility of the consumption of complementary products for good health. Therefore, the marginal utility of consumption may increase when health deteriorates.

Intergenerational norms may be another reason for the difference. Different from the US, the elderly in China receive more financial support from their children. When Chinese old people’s health deteriorates with age, such support from Children generally becomes stronger in order to make up their decreasing income and sustain their consumption level. In this way, the closer relationship between parents and children associated with parents’ deteriorating health may improve the marginal utility of consumption. Bequest motives may also explain the difference. Due to some institutional factors, Chinese old people generally have stronger motivation to leave a bequest to their children. Such increasingly stronger motivation along with deteriorating health makes the Chinese old people save more consumption, and thus pushes up the marginal utility of consumption.

#### 3.4.2. Extensions

We extend the baseline regression in the following aspects. First, we distinguish between symptomatic and asymptomatic chronic diseases, and include both of them into the regression. Table 4 shows the estimation results, in which the first column is the estimation result when cancer is classified as a symptomatic chronic disease, and the second column is where cancer is classified as an asymptomatic chronic disease.

As the results in the first and second columns of Table 4 are similar, we use the results when cancer is classified as a symptomatic disease as an example to discuss. According to the first column of Table 4, for both Samples 50 and 65, the coefficients of interaction terms of asymptomatic chronic diseases and annual household income are 0.005 and 0.006, respectively; statistically significant at 1% and 5%, respectively. These results show the positive effect of the increasing number of asymptomatic chronic diseases on the marginal utility of consumption. However, the coefficients of interaction terms of symptomatic chronic diseases and annual household income are not statistically significant at 10% significance level. The essential reason why the number of asymptomatic chronic diseases significantly affects the marginal utility of consumption is that asymptomatic chronic diseases impose fewer constraints for the non-medical consumption, for example, scientific exercise programs. As a result, one more asymptomatic chronic disease may incentivize individuals more significantly to consume in order to ameliorate health status. Next, we use whether an individual has a type of chronic disease to be the proxy of health status. For example, when we use hypertension as the proxy of health status, if an individual suffers from hypertension, then the proxy of health status “SOME CHRONIC DISEASE” equals 1, and 0 otherwise. Estimation results are shown in Table 5 and Table 6.

According to Table 5 and Table 6, for Sample 50, when an individual has one of the following chronic diseases: dyslipidemia, liver disease, heart attack, and kidney disease, the coefficient of the interaction term is positive and statistically significant, implying that the marginal utility of consumption is higher when an individual suffers from the chronic diseases mentioned above. For Sample 65, when an individual suffers from diabetes or stroke, the coefficient of the interaction term is positive and statistically significant.

In addition, we divide both Samples 50 and 65 into two sub-samples, that is, individuals who received education and never received education. Estimation results in Table 7 show that for subsamples in which individuals received education, $\beta_{1}$ is positive and statistically significant at 1% or 10%, implying that for individuals who received education, when their health status deteriorates, the marginal utility of consumption will increase. First, individuals who have received a good education usually have a higher degree of health consciousness and a stronger willingness to pay for good health. Accordingly, the deterioration of health status may incentivize these individuals to invest more in their health. Second, individuals who have received a good education usually have a higher income, and thus their looser budget constraints may allow them to consume more to improve their health.

Finally, we focus on an individual’s physical condition, which is one aspect of health status. We use three measures ADL, IADL and OFL to be the proxies of health status. Table 8 gives the estimation results. For Sample 50, all $\beta_{1}$ are negative and statistically significant at 10% or even lower significance level, implying that the marginal utility of non-medical consumption declines with the loss of individual physical function. However, for Sample 65, only when IADL is the health proxy, $\beta_{1}$ is negative and statistically significant. When the physical function of an individual gradually weakens, the marginal utility of the consumption of complementary products for good health dominates that of substitute products. Then, the individuals’ marginal utility of consumption decreases, and they may consume less non-medical products.

We have seen that the effects of health status on the marginal utility of consumption are opposites when we use the number of chronic diseases and physical conditions as health proxies. In fact, physical conditions can only reflect one aspect of health status, while the number of chronic diseases can comprehensively measure the health status to a higher degree. For example, the chronic diseases considered in this paper include both the type of disease that affects the physical condition (such as arthritis, diabetes and stroke), as well as heart disease, hypertension, stomach disease, liver disease, and kidney disease that may cause long-term damage to health conditions but impose fewer limitations on movement. It is natural to expect that if an individual finds movement difficult, her/his marginal utility of consumption may significantly decrease. However, if the health condition becomes worse but does not limit physical movements, she/he may have stronger incentives to consume products; for example, healthy meals, in order to improve health status.

#### 3.4.3. Robustness

We also conduct a series of robustness analyses. First, as psychiatric disease may affect the reporting of subjective well-being [23,38], we exclude it in the number of chronic diseases in the baseline regression. According to Table 9, we include the psychiatric disease and find that the results of baseline regression are robust.

Second, hukou is a system of household registration in China, which officially identifies an individual as a resident of a certain area. As hukou can reflect the differences between urban and rural areas to some extent, and is closely related to medical insurance, pension and employment, we also include the variable HUKOU in the regression. As we can see in Table 10, the estimation results of baseline regression and the regression including HUKOU are similar. Consequently, the results of baseline regression are robust to the inclusion of hukou. Moreover, both the coefficients of HUKOU of Samples 50 and 65 are positive and statistically significant at 1% level, indicating that individuals with non-agriculture hukou on average have higher utility than those with agriculture hukou. Currently, although the interests attached with hukou have been reduced significantly, the HUKOU variable can still show some differences between urban and rural areas, for example, different quality of education, medical services and infrastructure. There coefficients estimated in fact confirm the urban-rural difference not only in China but also in many other low- and middle-income countries.

Third, we use the annual household non-labor income, the average across three waves of annual household income, the average across three waves of annual household non-labor income, education, and residential location as proxies of consumption. The estimation results shown in Table 11 are generally consistent with the baseline regression, especially for Sample 50. Note that for Sample 65, the coefficient of the interaction term between chronic disease and consumption is no longer significant when we follow exactly the relevant study in the US. Such results may be due to the fact that non-labor income may not be an ideal proxy for consumption in China and the income of Chinese old people varies relatively more significantly across years. Furthermore, we also use CESD8 and CESD4 as proxies of the utility variable. Estimation results shown in Table 12 imply that when the health status deteriorates, an individual’s marginal utility of non-medical consumption will increase, which is also consistent with insights from the baseline regression.

Finally, we use the same dependent and explanatory variables as in the baseline regression but estimate by logit and probit models. Table 13 gives the average marginal effect, in which robust standard errors and cluster-robust standard errors are calculated. Estimation results shown in Table 13 imply similar insights as in the baseline regression.

## 4. Conclusions and Policy Implications

In this article, we investigate how the deteriorating health status of an individual affects the marginal utility of non-medical consumption in China. By using CHARLS 2011, 2013 and 2015 data and estimating a linear probability model, we find that when the number of chronic diseases increases one standard deviation, the marginal utility of consumption will increase by 16.0% and 20.0% for samples of the middle-aged and elderly individuals over 50 and 65 years of age, respectively. This result is contrary to some extent to the findings from the US [23]. Different economic development stages, intergenerational norms and bequest motives may be reasons for these contrasting patterns between China and the US.

Moreover, we extend the baseline regression by considering, for example, the symptom of chronic diseases, education and physical conditions. Our results are also robust to the inclusion of psychiatric disease and hukou (i.e., a system of household registration in China), various consumption and utility proxies, and the use of logit and probit models.

Our results have clear policy implications. Given the results of two simulation exercises (see Supplementary Materials file), we find that there may exist a possibility of underestimation of both the optimal reimbursement ratio of medical insurance and saving rate if we do not consider the effect of health on the marginal utility of consumption. Therefore, realizing the effect of health identified in this paper may be helpful for China to correct the possible bias in the current reimbursement policy of medical insurance. In addition, realizing such effect may also be conducive to a better understanding of optimal saving rate, which would benefit the scientific evaluation of saving, investment and economic growth in China.

## Figures and Tables

**Table 1:** Summary statistics of variables.

**Panel A:**		**Sample 50: Age ≥ 50**				
**Type**	**Variable**	**Obs.**	**Mean**	**S.D.**	**Min**	**Max**
Utility	HAPPY	37856	0.416	0.493	0	1
	CESD8	41176	4.345	2.433	0	8
	CESD4	41176	2.151	1.276	0	4
Health	CHRONIC DISEASE	37856	1.279	1.364	0	10
	ADL	37856	0.366	0.934	0	6
	IADL	37856	0.226	0.594	0	3
	OFL	37856	2.109	2.154	0	9
Consumption	HOUSEHOLD INCOME (unit: Yuan)	37856	29591.23	33934.97	0.075	207170
	HOUSEHOLD NL INCOME (unit: Yuan)	37718	12726.48	18270.85	0.075	113300
	AVE HOUSEHOLD INCOME (unit: Yuan)	38191	28350.89	27117.15	148.328	165508.1
	AVE HOUSEHOLD NL INCOME (unit: Yuan)	38221	12037.05	14901.04	25	90567.57
	EDUCATION	38218	0.682	0.466	0	1
	RESIDENTIAL LOCATION	23666	0.385	0.487	0	1
Individual	FEMALE	37856	0.509	0.5	0	1
	AGE	37856	62.421	8.572	50	102
	MARRIED	37856	0.857	0.35	0	1
Household	HOUSEHOLD SIZE	37856	3.417	1.79	1	16
	HUKOU	37856	0.270	0.444	0	1
**Panel B:**		**Sample 65: ≥ 65**				
**Type**	**Variable**	**Obs.**	**Mean**	**S.D.**	**Min**	**Max**
Utility	HAPPY	13594	0.412	0.492	0	1
	CESD8	15133	4.109	2.475	0	8
	CESD4	15133	2.022	1.283	0	4
Health	CHRONIC DISEASE	13594	1.456	1.419	0	10
	ADL	13594	0.560	1.144	0	6
	IADL	13594	0.354	0.732	0	3
	OFL	13594	2.767	2.350	0	9
Consumption	HOUSEHOLD INCOME (unit: Yuan)	13594	23532.92	28070.06	0.45	166856.5
	HOUSEHOLD NL INCOME (unit: Yuan)	13606	15729.64	21043.76	0.45	122550
	AVE HOUSEHOLD INCOME (unit: Yuan)	13670	22658.93	22697.92	245	132431.3
	AVE HOUSEHOLD NL INCOME (unit: Yuan)	13668	14928.26	17749.26	237.916	101741.2
	EDUCATION	13705	0.602	0.490	0	1
	RESIDENTIAL LOCATION	8297	0.380	0.485	0	1
Individual	FEMALE	13594	0.489	0.500	0	1
	AGE	13594	71.828	5.647	65	102
	MARRIED	13594	0.742	0.437	0	1
Household	HOUSEHOLD SIZE	13594	3.091	1.708	1	15
	HUKOU	13594	0.293	0.455	0	1

**Table 2:** Estimation results.

Estimation Method:	Baseline		LPM		LPM (Robust S.E.)	
	LPM (Robust S.E.)		(Cluster-Robust S.E.)		(& Provincial FE)	
Dependent Variable: HAPPY	Age ≥ 50	Age ≥ 65	Age ≥ 50	Age ≥ 65	Age ≥ 50	Age ≥ 65
CHRONIC DISEASE × ln(HOUSEHOLD INCOME) (*β*_1_)	0.003 ***	0.004 **	0.003 ***	0.004 **	0.003 ***	0.004 **
	(0.001)	(0.002)	(0.001)	(0.002)	(0.001)	(0.002)
CHRONIC DISEASE (*β*_3_)	−0.072 ***	−0.080 ***	−0.072 ***	−0.080 ***	−0.072 ***	−0.078 ***
	(0.011)	(0.019)	(0.011)	(0.019)	(0.011)	(0.019)
ln(HOUSEHOLD INCOME) (*β*_4_)	0.027 ***	0.030 ***	0.027 ***	0.030 ***	0.026 ***	0.030 ***
	(0.002)	(0.004)	(0.002)	(0.004)	(0.002)	(0.004)
FEMALE	−0.029 ***	−0.026 ***	−0.029 ***	−0.026 ***	−0.030 ***	−0.029 ***
	(0.005)	(0.009)	(0.006)	(0.010)	(0.005)	(0.009)
AGE	−0.000	0.041 ***	−0.000	0.041 **	0.001	0.043 ***
	(0.004)	(0.016)	(0.004)	(0.016)	(0.004)	(0.016)
AGE^2^	0.000	−0.000**	0.000	−0.000**	0.000	−0.000***
	(0.000)	(0.000)	(0.000)	(0.000)	(0.000)	(0.000)
MARRIED	0.065 ***	0.039 ***	0.065 ***	0.039 ***	0.060 ***	0.034 ***
	(0.007)	(0.010)	(0.008)	(0.011)	(0.007)	(0.010)
HOUSEHOLD SIZE	−0.021 ***	−0.026 ***	−0.021 ***	−0.026 ***	−0.018 ***	−0.024 ***
	(0.001)	(0.002)	(0.001)	(0.003)	(0.001)	(0.003)
Constant	0.210 *	−1.316 **	0.210	−1.316 **	0.195	−1.362 **
	(0.121)	(0.584)	(0.132)	(0.608)	(0.123)	(0.588)
Obs.	37856	13594	37856	13594	37856	13594
Adjusted R^2^	0.032	0.032	0.032	0.032	0.040	0.039

Note: Robust standard errors in parentheses; *, ** and *** represent that coefficients are statistically significant at 10%, 5% and 1%, respectively.

**Table 3:** Effect of health status on marginal utility of consumption.

	Baseline	
Dependent Variable: HAPPY	Age ≥ 50	Age ≥ 65
**Panel A: Percentage change in marginal utility of consumption**		
CHRONIC DISEASE increases by 1 unit, percentage change in marginal utility ($\beta_{1}/\beta_{4}$)	11.7%	14.1%
Bootstrapped Standard Error	5.1%	8.6%
(Bootstrapped 95% Confidence Interval)	(1.6%, 21.7%)	(−2.7%, 30.9%)
[Bootstrapped p-value]	[0.023]	[0.100]
CHRONIC DISEASE increases by 1 s.d., percentage change in marginal utility ($\sigma_{health}\beta_{1}/\beta_{4}$)	16.0%	20.0%
Bootstrapped Standard Error	7.0%	12.2%
(Bootstrapped 95% Confidence Interval)	(2.2%, 29.6%)	(−3.8%, 43.8%)
[Bootstrapped p-value]	[0.023]	[0.100]
**Panel B: Absolute change in marginal utility of consumption**		
Marginal Utility of Consumption (0 disease)	0.027	0.030
Marginal Utility of Consumption (1 disease)	0.030	0.034
Marginal Utility of Consumption (2 diseases)	0.033	0.038
Marginal Utility of Consumption (3 diseases)	0.036	0.042
Marginal Utility of Consumption (4 diseases)	0.039	0.046
Marginal Utility of Consumption (5 diseases)	0.042	0.050
6 and more diseases		

Note: Bootstrapped standard errors, 95% confidence intervals and p-values are based on 1000 bootstrap iterations, resampling individuals with replacement; the seed is 3.

**Table 4:** By symptomatic and asymptomatic chronic diseases.

	Cancer is Symptomatic		Cancer is Asymptomatic	
Dependent Variable: HAPPY	Age ≥ 50	Age ≥ 65	Age ≥ 50	Age ≥ 65
SYMPTOMATIC CHRONIC DISEASE × ln(HOUSEHOLD INCOME)	−0.002	−0.001	−0.002	−0.000
	(0.003)	(0.004)	(0.003)	(0.004)
ASYMPTOMATIC CHRONIC DISEASE × ln(HOUSEHOLD INCOME)	0.005 ***	0.006 **	0.004 ***	0.005 **
	(0.002)	(0.003)	(0.001)	(0.003)
SYMPTOMATIC CHRONIC DISEASE	−0.056 **	−0.055	−0.061 **	−0.062
	(0.024)	(0.040)	(0.025)	(0.041)
ASYMPTOMATIC CHRONIC DISEASE	−0.072 ***	−0.083 ***	−0.069 ***	−0.079 ***
	(0.015)	(0.025)	(0.014)	(0.025)
ln(HOUSEHOLD INCOME)	0.027 ***	0.030 ***	0.027 ***	0.030 ***
	(0.002)	(0.004)	(0.002)	(0.004)
Obs.	37856	13594	37856	13594
Adjusted R^2^	0.034	0.033	0.034	0.033

Notes: (1) Robust standard errors in parentheses; (2) *, ** and *** represent that coefficients are statistically significant at 10%, 5% and 1%, respectively; (3) Coefficients of other explanatory variables are not reported.

**Table 5:** Effect of a chronic disease on marginal utility of consumption (Sample 50).

Dependent Variable: HAPPY	Sample 50: Age ≥ 50				
	**Hypertension**	**Dyslipidemia**	**Diabetes**	**Cancer**	
SOME CHRONIC DISEASE	0.005	0.014 ***	0.010	−0.015	
× ln(HOUSEHOLD INCOME) (*β*_1_)					
	(0.004)	(0.005)	(0.007)	(0.018)	
CHRONIC DISEASE (*β*_3_)	−0.069 **	−0.147 ***	−0.129 **	0.116	
	(0.035)	(0.050)	(0.065)	(0.177)	
ln (HOUSEHOLD INCOME) (*β*_4_)	0.029 ***	0.029 ***	0.030 ***	0.031 ***	
	(0.002)	(0.002)	(0.002)	(0.002)	
Obs.	37856	37856	37856	37856	
Adjusted R^2^	0.018	0.018	0.018	0.018	
MUC (0 disease)	0.029	0.029	0.030	0.031	
MUC (1 disease)	0.034	0.043	0.040	0.016	
	**Lung disease**	**Liver disease**	**Heart attack**	**Stroke**	
SOME CHRONIC DISEASE	−0.002	0.014 *	0.013 ***	0.005	
× ln(HOUSEHOLD INCOME) (*β*_1_)					
	(0.006)	(0.008)	(0.005)	(0.010)	
CHRONIC DISEASE (*β*_3_)	−0.075	−0.222 ***	−0.190 ***	−0.131	
	(0.054)	(0.079)	(0.045)	(0.089)	
ln(HOUSEHOLD INCOME) (*β*_4_)	0.030 ***	0.030 ***	0.029 ***	0.030 ***	
	(0.002)	(0.002)	(0.002)	(0.002)	
Obs.	37856	37856	37856	37856	
Adjusted R^2^	0.021	0.019	0.020	0.018	
MUC (0 disease)	0.030	0.030	0.029	0.030	
MUC (1 disease)	0.028	0.044	0.042	0.035	
	**Kidney disease**	**Stomach disease**	**Memory-related disease**	**Arthritis**	**Asthma**
SOME CHRONIC DISEASE	0.015 **	0.002	−0.023 *	0.003	−0.008
× ln(HOUSEHOLD INCOME) (*β*_1_)					
	(0.007)	(0.004)	(0.013)	(0.004)	(0.009)
CHRONIC DISEASE (*β*_3_)	−0.260 ***	−0.124 ***	0.085	−0.147 ***	−0.002
	(0.068)	(0.037)	(0.127)	(0.033)	(0.088)
ln(HOUSEHOLD INCOME) (*β*_4_)	0.030 ***	0.029 ***	0.031 ***	0.028 ***	0.031 ***
	(0.002)	(0.002)	(0.002)	(0.002)	(0.002)
Obs.	37856	37856	37856	37856	37856
Adjusted R^2^	0.021	0.025	0.019	0.030	0.018
MUC (0 disease)	0.030	0.029	0.031	0.028	0.031
MUC (1 disease)	0.045	0.031	0.008	0.031	0.023

Notes: (1) Robust standard errors in parentheses; (2) *, ** and *** represent that coefficients are statistically significant at 10%, 5% and 1%, respectively; (3) The variable “SOME CHRONIC DISEASE” indicate whether an individual has some kind of chronic disease; (4) MUC is the abbreviation of the marginal utility of consumption; (5) Coefficients of other explanatory variables are not reported.

**Table 6:** Effect of a chronic disease on marginal utility of consumption (Sample 65).

Dependent Variable: HAPPY	Sample 65: Age ≥ 65				
	**Hypertension**	**Dyslipidemia**	**Diabetes**	**Cancer**	
SOME CHRONIC DISEASE	0.005	0.014	0.031 ***	−0.024	
× ln(HOUSEHOLD INCOME) (*β*_1_)					
	(0.006)	(0.009)	(0.011)	(0.034)	
CHRONIC DISEASE (*β*_3_)	−0.069	−0.132	−0.352 ***	0.159	
	(0.058)	(0.088)	(0.104)	(0.344)	
ln (HOUSEHOLD INCOME) (*β*_4_)	0.033 ***	0.033 ***	0.033 ***	0.034 ***	
	(0.004)	(0.003)	(0.003)	(0.003)	
Obs.	13594	13594	13594	13594	
Adjusted R^2^	0.019	0.018	0.019	0.018	
MUC (0 disease)	0.033	0.033	0.033	0.034	
MUC (1 disease)	0.038	0.047	0.064	0.010	
	**Lung disease**	**Liver disease**	**Heart attack**	**Stroke**	
SOME CHRONIC DISEASE	−0.010	0.020	0.009	0.027 *	
× ln(HOUSEHOLD INCOME) (*β*_1_)					
	(0.009)	(0.015)	(0.008)	(0.014)	
CHRONIC DISEASE (*β*_3_)	0.012	−0.292 **	−0.145 **	−0.324 **	
	(0.084)	(0.147)	(0.073)	(0.135)	
ln(HOUSEHOLD INCOME) (*β*_4_)	0.035 ***	0.034 ***	0.034 ***	0.033 ***	
	(0.003)	(0.003)	(0.003)	(0.003)	
Obs.	13594	13594	13594	13594	
Adjusted R^2^	0.022	0.020	0.020	0.019	
MUC (0 disease)	0.035	0.034	0.034	0.033	
MUC (1 disease)	0.025	0.054	0.043	0.060	
	**Kidney disease**	**Stomach disease**	**Memory-related disease**	**Arthritis**	**Asthma**
SOME CHRONIC DISEASE	0.014	−0.002	−0.033 *	0.005	−0.001
× ln(HOUSEHOLD INCOME) (*β*_1_)					
	(0.014)	(0.008)	(0.018)	(0.006)	(0.014)
CHRONIC DISEASE (*β*_3_)	−0.253 *	−0.091	0.183	−0.155 ***	−0.049
	(0.132)	(0.070)	(0.170)	(0.060)	(0.128)
ln(HOUSEHOLD INCOME) (*β*_4_)	0.034 ***	0.033 ***	0.035 ***	0.031 ***	0.034 ***
	(0.003)	(0.003)	(0.003)	(0.003)	(0.003)
Obs.	13594	13594	13594	13594	13594
Adjusted R^2^	0.022	0.025	0.020	0.028	0.019
MUC (0 disease)	0.034	0.033	0.035	0.031	0.034
MUC (1 disease)	0.048	0.031	0.002	0.036	0.033

Notes: (1) Robust standard errors in parentheses; (2) *, ** and *** represent that coefficients are statistically significant at 10%, 5% and 1%, respectively; (3) The variable “SOME CHRONIC DISEASE” indicate whether an individual has some kind of chronic disease; (4) MUC is the abbreviation of the marginal utility of consumption; (5) Coefficients of other explanatory variables are not reported.

**Table 7:** By education.

	Age ≥ 50		Age ≥ 65	
Dependent Variable: HAPPY	No Education	Education	No Education	Education
CHRONIC DISEASE × ln(HOUSEHOLD INCOME) (*β*_1_)	−0.002	0.004 ***	0.000	0.005 *
	(0.002)	(0.001)	(0.004)	(0.003)
CHRONIC DISEASE (*β*_3_)	−0.029	−0.084 ***	−0.049	−0.084 ***
	(0.020)	(0.013)	(0.033)	(0.024)
ln(HOUSEHOLD INCOME) (*β*_4_)	0.023 ***	0.028 ***	0.030 ***	0.032 ***
	(0.004)	(0.003)	(0.007)	(0.006)
Obs.	11998	25858	5364	8229
Adjusted R^2^	0.031	0.030	0.028	0.033

Notes: (1) Robust standard errors in parentheses; (2) *, ** and *** represent that coefficients are statistically significant at 10%, 5% and 1%, respectively; (3) Coefficients of other explanatory variables are not reported.

**Table 8:** Health by physical condition.

PHYSICAL CONDITION:	ADL		IADL		OFL	
Dependent Variable: HAPPY	Age ≥ 50	Age ≥ 65	Age ≥ 50	Age ≥ 65	Age ≥ 50	Age ≥ 65
PHYSICAL CONDITION × ln(HOUSEHOLD INCOME) (*β*_1_)	−0.003 *	−0.003	−0.004 *	−0.006 *	−0.001 **	−0.002
	(0.002)	(0.002)	(0.002)	(0.003)	(0.001)	(0.001)
PHYSICAL CONDITION (*β*_3_)	−0.043 ***	−0.034	−0.062 ***	−0.034	−0.033 ***	−0.029 ***
	(0.014)	(0.022)	(0.019)	(0.029)	(0.006)	(0.010)
ln(HOUSEHOLD INCOME) (*β*_4_)	0.028 ***	0.031 ***	0.028 ***	0.032 ***	0.025 ***	0.029 ***
	(0.002)	(0.003)	(0.002)	(0.003)	(0.002)	(0.004)
Obs.	37856	13594	37856	13594	37856	13594
Adjusted R^2^	0.034	0.039	0.031	0.035	0.053	0.056

Notes: (1) Robust standard errors in parentheses; (2) *, ** and *** represent that coefficients are statistically significant at 10%, 5% and 1%, respectively; (3) Coefficients of other explanatory variables are not reported.

**Table 9:** Including psychiatric disease.

	Baseline		Considering Psychiatric Disease	
Dependent Variable: HAPPY	Age ≥ 50	Age ≥ 65	Age ≥ 50	Age ≥ 65
CHRONIC DISEASE × ln(HOUSEHOLD INCOME) (*β*_1_)	0.003 ***	0.004 **	0.003 ***	0.004 **
	(0.001)	(0.002)	(0.001)	(0.002)
CHRONIC DISEASE (*β*_3_)	−0.072 ***	−0.080 ***	−0.073 ***	−0.082 ***
	(0.011)	(0.019)	(0.011)	(0.018)
ln(HOUSEHOLD INCOME) (*β*_4_)	0.027 ***	0.030 ***	0.027 ***	0.030 ***
	(0.002)	(0.004)	(0.002)	(0.004)
Obs.	37856	13594	38167	13707
Adjusted R^2^	0.032	0.032	0.032	0.033

Notes: (1) Robust standard errors in parentheses; (2) *, ** and *** represent that coefficients are statistically significant at 10%, 5% and 1%, respectively; (3) Coefficients of other explanatory variables are not reported.

**Table 10:** With HUKOU variable.

	Baseline		HUKOU	
Dependent Variable: HAPPY	Age ≥ 50	Age ≥ 65	Age ≥ 50	Age ≥ 65
CHRONIC DISEASE × ln(HOUSEHOLD INCOME) (*β*_1_)	0.003 ***	0.004 **	0.002 **	0.003 *
	(0.001)	(0.002)	(0.001)	(0.002)
CHRONIC DISEASE (*β*_3_)	−0.072 ***	−0.080 ***	−0.066 ***	−0.072 ***
	(0.011)	(0.019)	(0.011)	(0.018)
ln(HOUSEHOLD INCOME) (*β*_4_)	0.027 ***	0.030 ***	0.022 ***	0.023 ***
	(0.002)	(0.004)	(0.002)	(0.004)
FEMALE	−0.029 ***	−0.026 ***	−0.029 ***	−0.026 ***
	(0.005)	(0.009)	(0.005)	(0.009)
AGE	−0.000	0.041 ***	−0.000	0.038 **
	(0.004)	(0.016)	(0.004)	(0.016)
AGE^2^	0.000	−0.000 **	0.000	−0.000 **
	(0.000)	(0.000)	(0.000)	(0.000)
MARRIED	0.065 ***	0.039 ***	0.062 ***	0.035 ***
	(0.007)	(0.010)	(0.007)	(0.010)
HOUSEHOLD SIZE	−0.021 ***	−0.026 ***	−0.017 ***	−0.022 ***
	(0.001)	(0.002)	(0.001)	(0.003)
HUKOU			0.080 ***	0.082 ***
			(0.006)	(0.010)
Constant	0.210 *	−1.316 **	0.235 *	−1.150 **
	(0.121)	(0.584)	(0.121)	(0.584)
Obs.	37856	13594	37856	13594
Adjusted R^2^	0.032	0.032	0.036	0.037

Notes: (1) Robust standard errors in parentheses; (2) *, ** and *** represent that coefficients are statistically significant at 10%, 5% and 1%, respectively.

**Table 11:** Consumption proxies.

**CONSUMPTION:**	**HOUSEHOLD**		**AVE HOUSEHOLD**		**AVE HOUSEHOLD**	
	**NL INCOME**		**INCOME**		**NL INCOME**	
**Dependent Variable: HAPPY**	**Age ≥ 50**	**Age ≥ 65**	**Age ≥ 50**	**Age ≥ 65**	**Age ≥ 50**	**Age ≥ 65**
CHRONIC DISEASE × ln(CONSUMPTION) (*β*_1_)	0.003 ***	0.003	0.003 *	0.003	0.004 ***	0.000
	(0.001)	(0.002)	(0.002)	(0.003)	(0.001)	(0.002)
CHRONIC DISEASE (*β*_3_)	−0.074 ***	−0.069 ***	−0.066 ***	−0.069 ***	−0.080 ***	−0.042 *
	(0.009)	(0.017)	(0.015)	(0.025)	(0.012)	(0.022)
ln(CONSUMPTION) (*β*_4_)	0.015 ***	0.025 ***	0.053 ***	0.055 ***	0.032 ***	0.053 ***
	(0.002)	(0.004)	(0.003)	(0.006)	(0.003)	(0.005)
Obs.	37718	13606	38191	13670	38221	13668
Adjusted R^2^	0.027	0.029	0.043	0.041	0.038	0.039
**CONSUMPTION:**	**EDUCATION**		**RESIDENTIAL LOCATION**			
**Dependent Variable: HAPPY**	**Age ≥ 50**	**Age ≥ 65**	**Age ≥ 50**	**Age ≥ 65**		
CHRONIC DISEASE × ln(CONSUMPTION) (*β*_1_)	0.009 **	0.009	0.010 **	0.014 *		
	(0.004)	(0.006)	(0.004)	(0.007)		
CHRONIC DISEASE (*β*_3_)	−0.050 ***	−0.046 ***	−0.043 ***	−0.037 ***		
	(0.003)	(0.005)	(0.003)	(0.004)		
ln(CONSUMPTION) (*β*_4_)	0.034 ***	0.035 ***	0.089 ***	0.088 ***		
	(0.008)	(0.013)	(0.009)	(0.017)		
Obs.	38218	13705	23666	8297		
Adjusted R^2^	0.023	0.022	0.026	0.025		

Notes: (1) Robust standard errors in parentheses; (2) *, ** and *** represent that marginal effects are statistically significant at 10%, 5% and 1%, respectively; (3) Coefficients of other explanatory variables are not reported.

**Table 12:** Utility proxies.

Dependent Variable:	CESD8		CESD4	
	Age ≥ 50	Age ≥ 65	Age ≥ 50	Age ≥ 65
CHRONIC DISEASE × ln(HOUSEHOLD INCOME) (*β*_1_)	0.030 ***	0.031 ***	0.013 ***	0.014 ***
	(0.005)	(0.009)	(0.003)	(0.005)
CHRONIC DISEASE (*β*_3_)	−0.578 ***	−0.581 ***	−0.236 ***	−0.244 ***
	(0.052)	(0.090)	(0.028)	(0.047)
ln(HOUSEHOLD INCOME) (*β*_4_)	0.171 ***	0.244 ***	0.081 ***	0.109 ***
	(0.010)	(0.020)	(0.005)	(0.010)
Obs.	41176	15133	41176	15133
Adjusted R^2^	0.083	0.117	0.062	0.090

*Notes*: (1) Robust standard errors in parentheses; (2) *, ** and *** represent that coefficients are statistically significant at 10%, 5% and 1%, respectively; (3) Coefficients of other explanatory variables are not reported.

**Table 13:** Effects from discrete choice models.

Estimation Method:	Logit		Logit		Probit	
	(Robust S.E.)		(Cluster-Robust S.E.)		(Cluster-Robust S.E.)	
Dependent Variable: HAPPY	Age ≥ 50	Age ≥ 65	Age ≥ 50	Age ≥ 65	Age ≥ 50	Age ≥ 65
CHRONIC DISEASE × ln(HOUSEHOLD INCOME)	0.0057 ***	0.0065 **	0.0057 ***	0.0065 **	0.0047 ***	0.0057 **
CHRONIC DISEASE	−0.0990 ***	−0.1031 ***	−0.0990 ***	−0.1031 ***	−0.0884 ***	−0.0945 ***
ln(HOUSEHOLD INCOME)	0.0255 ***	0.0292 ***	0.0255 ***	0.0291 ***	0.0256 ***	0.0289 ***
FEMALE	−0.0281 ***	−0.0257 ***	−0.0281 ***	−0.0257 **	−0.0282 ***	−0.0258 **
AGE	−0.0004	0.0413 ***	0.0004	0.0413 **	0.0002	0.0408 **
AGE^2^	6.78×10^-6^	−0.0003 **	6.78×10^-6^	−0.0003 **	8.26×10^-6^	−0.0003 **
MARRIED	0.0659 ***	0.0394 ***	0.0659 ***	0.0394 ***	0.0655 ***	0.0387 ***
HOUSEHOLD SIZE	−0.0212 ***	−0.0270 ***	−0.0212 ***	−0.0270 ***	−0.0209 ***	−0.0264 ***
Obs.	37856	13594	37856	13594	37856	13594

Note: 1. Standard errors in parentheses; 2. *, ** and *** represent that marginal effects are statistically significant at 10%, 5% and 1%, respectively. 3. The numbers shown in Table 13 is the average marginal effect.

## Supplementary Materials

The following are available online at https://www.mdpi.com/1660-4601/17/7/2234/s1, S.1: Sources of household income in CHARLS, S.2: Facts on medical insurance in China, S.3: Policy applications: Optimal reimbursement ratio of medical insurance and saving rate.
