# Supplementary material for: How Does Health Status Affect Marginal Utility of Consumption? Evidence from China
Source: Int J Environ Res Public Health. 2020 Mar 26;17(7):2234. doi: 10.3390/ijerph17072234 (PMC7177955; doi:10.3390/ijerph17072234)
Supplement: Supplementary file 1 [file ijerph-17-02234-s001.pdf]

How Does Health Status Affect Marginal Utility of Consumption? Evidence from China

Supplementary Materials File

S.1. Sources of household income in CHARLS

Table S.1: Sources of household income

Categories		Income sources
Non-labor income	Pension	Monthly pension
		Supplementary pension insurance of the firms
		Commercial pension benefits
		Rural pension benefits
		Pension subsidy for the oldest old
		Other pension programs
		New rural social pension insurance
	Private transfer	Transfer from non-coresident parents
		Transfer from non-coresident parents-in-law
		Transfer from children
		Transfer from grandchildren
		Transfer from non-coresident other relatives
		Transfer from non-coresident non-relatives
	Public transfer	Public transfer for low-income household
		Other government subsidies
		Donation and compensation
Labor income		After-tax salary (including bonus)
		Agricultural net profit
		Revenue from self-employed activities
		Income from side jobs other than main jobs
Financial assets		Cash at home
		Deposits in financial institutions
		Government bonds
		Market value of stock
		Market value of mutual funds
		Public housing funds
		Jizikuan (fund-raising in some organizations)
		Unpaid salary
		Asset value under a person's name other than household members
		Benefits from other investments

S.2. Facts on medical insurance in China

The medical insurance policy in China is relatively stable. We use the most recent statics in 2018 to introduce (see the official website of National Healthcare Security Administration http://www.nhsa.gov.cn/).

The basic medical insurance in China has a high coverage rate. According to statistics in 2018, the participation rate of basic medical insurance exceeded 95%. The major schemes of basic medical insurance in China include the urban employee-based basic medical insurance scheme (UEBMI) and the urban and rural resident-based basic medical insurance scheme (URRBMI).

For UEBMI, the amount that firm employers pay is, on average, around 7% of the salary of employees, and the amount that employees pay is, on average, around 2% of the salary of employees.

In 2018, the reimbursement ratio under the UEBMI policy is 81.6%. The average of total medical expenditure across all UEBMI participants is 3313 CNY (around 476 USD). The average of total inpatient medical expenditure across the total number of inpatient medical visits is 11181 CNY (around 1607 USD). The average of out-of-pocket inpatient medical expenditure across the total number of inpatient medical visits is 3153 CNY (around 453 USD).

The medical insurance for Chinese residents before 2013 mainly includes the urban resident-based basic medical insurance scheme (URBMI) and the rural new cooperative medical scheme (NCMS). Starting from 2013, URBMI and NCMS gradually merge to URRBMI in each province of China. The source of URRBMI combines individual payment and government subsidy. In 2018, the average of individual payment across all URRBMI participants is 693 CNY (around 100 USD). The average of government subsidy across all URRBMI participants is 497 CNY (around 71 USD). The average of total medical expenditure across all URRBMI participants is 1183 CNY (around 170 USD). The average of total inpatient medical expenditure across the total number of inpatient medical visits is 6577 CNY (around 945 USD). The average of out-of-pocket inpatient medical expenditure across the total number of inpatient medical visits is 2887 CNY (around 415 USD).

In addition, the outpatient medical expenditure in UEBMI and URRBMI is supported by both individual account and public reimbursement. The reimbursement for treatments of chronic diseases in outpatient medical expenditure refers to the that of inpatient treatments.

Overall, Chinese individuals generally enjoy a relatively high reimbursement ratio of medical insurance and suffer from a relatively low out-of-pocket medical expenditure.

S.3. Policy applications: Optimal reimbursement ratio of medical insurance and saving rate

The effect of health insurance on the marginal utility of consumption plays an important role in the design of medical insurance and saving behaviour. Thus, by using the models in Finkelstein et al. (2008) and Chetty (2006), and parameter values estimated from some Chinese studies, we simulate the optimal reimbursement ratio of medical insurance and saving rate, considering the effect of health insurance on the marginal utility of consumption or not. These exercises will help us to understand the importance of such effect for health and even for the development of economy in China.

S.3.1. Optimal reimbursement ratio of medical insurance

First consider a theoretical model incorporating medical insurance. Individuals have probability p to be sick. If an individuals is sick, he/she will incur medical expenditure H, in which proportion b can be reimbursed by medical insurance. If an individual is healthy, he/she will pay an amount τ for medical insurance.

Given a permanent income Y, considering the budget constraints under the healthy and sick states, an individual maximizes utility:

$$\max_{C_h, C_s, H} (1-p)u_h(C_h) + p\big(u_s(C_s) + \Psi(H)\big), \tag{S.1}$$

$$s.t. Y - \tau - C_h \geq 0,$$

$$Y - C_s - (1-b)H \geq 0,$$

in which C_h and C_s are the amount of non-medical expenditure under the healthy and sick state, respectively; $u_h(C_h)$ and $u_s(C_s)$ are the utility under the healthy and sick state, respectively; and $\Psi(H)$ is the utility from medical services.

Under the budget balance constraint, that is, expected insurance payment equals to expected medical reimbursement, a social planner chooses b and τ to maximize individual utility:

$$\max_{b,\tau} V(b,\tau), \tag{S.2}$$

$$s.t.\ (1-p)\tau = pbH.$$

Focusing on interior solution, the optimal reimbursement needs to satisfy:

$$\left.\frac{dV}{db}\right|_{b=b^*} = 0. \tag{S.3}$$

In order to compute dV/db, we can solve the following Lagrangian function $V(b)$:

$$V(b) = \max_{C_h, C_s, H, \lambda_h, \lambda_s} (1-p)u_h(C_h) + p\big(u_s(C_s) + \Psi(H)\big) + \lambda_h\,(Y - \tau - C_h),$$

$$+\lambda_s(Y - C_s - (1-b)H), \tag{S.4}$$

in which λ_h and λ_s are Lagrangian multipliers. According to the Envelope Theorem, we have:

$$\left.\frac{dV}{db}\right|_{b=b^*} = -\lambda_h \frac{d\tau}{db} + \lambda_s H = 0$$

$$\Rightarrow \lambda_h \frac{d\tau}{db} = \lambda_s H \tag{S.5}$$

Taking the first-order condition of $V(b)$ with respect to λ_h and λ_s, we can obtain:

$$\lambda_h = (1-p)u_h'(C_h), \tag{S.6}$$

$$\lambda_s = pu_s'(C_s), \tag{S.7}$$

The budget balance of social planner gives:

$$\frac{d\tau}{db} = \frac{p}{1-p}\left(H + b\frac{dH}{db}\right). \tag{S.8}$$

Plugging λ_h, λ_s and $d\tau/db$ into $dV/db = 0$, we can obtain:

$$u_h'(C_h)\left(1 + \frac{b}{H}\frac{dH}{db}\right) = u_s'(C_s)$$

$$\Rightarrow u_h'(C_h)\left(1 + \frac{d\ln H}{d\ln b}\right) = u_s'(C_s)$$

$$\Rightarrow u_h'(C_h)\big(1 + \varepsilon_{H,b}\big) = u_s'(C_s) \tag{S.9}$$

Thus, the solution for the optimal reimbursement ratio of medical insurance is given by:

$$\frac{u_s'(C_s) - u_h'(C_h)}{u_h'(C_h)} = \varepsilon_{H,b}, \tag{S.10}$$

in which $\varepsilon_{H,b}$ is the reimbursement ratio elasticity of medical expenditure, measuring that the percentage change in medical expenditure in response to one percent increase in reimbursement ratio.

When we consider the possible effect of health on the marginal utility of consumption, we assume that the marginal utility of consumption in the sick state is $u_s'(C) = (1+\varphi_1)u_h'(C) = (1+\varphi_1)u'(C)$. Suppose a CRRA utility function:

$$u(C) = \frac{1}{1-\gamma}C^{1-\gamma} \tag{S.11}$$

Then, we can solve the optimal reimbursement ratio of medical insurance b^*:

$$\frac{(1+\varphi_1)(C_h - (1-b^*)H)^{-\gamma} - C_h^{-\gamma}}{C_h^{-\gamma}} = \varepsilon_{H,b}$$

$$\Rightarrow (C_h - (1 - b^*)H)^{-\gamma} = \frac{\varepsilon_{H,b} C_h^{-\gamma} + C_h^{-\gamma}}{1 + \varphi_1}$$

$$\Rightarrow (1 - b^*)H = C_h - \left[\frac{(1 + \varepsilon_{H,b}) C_h^{-\gamma}}{1 + \varphi_1}\right]^{\frac{-1}{\gamma}}$$

$$\Rightarrow 1 - b^* = \frac{C_h}{H} - \left(\frac{1 + \varphi_1}{1 + \varepsilon_{H,b}}\right)^{\frac{1}{\gamma}} \frac{C_h}{H}$$

$$\Rightarrow b^* = \left[\left(\frac{1+\varphi_1}{1+\varepsilon_{H,b}}\right)^{\frac{1}{\gamma}} - 1\right] \frac{C_h}{H} + 1 \quad \text{(S.12)}$$

The simulation for the value of b^* needs the following parameter values.

a) The effect of health status on the marginal utility of consumption φ_1. We use the estimation results for Sample 50 in the baseline regression ($\varphi_1 = \beta_1/\beta_4 = 11.7\%$).

b) The reimbursement ratio elasticity of medical expenditure $\varepsilon_{H,b}$. According to Bao and Zhao (2017), when the reimbursement ratio of rural resident increases by 1%, the inpatient medical expenditure will increase by 0.34%. Thus, in the simulation, we consider $\varepsilon_{H,b} = 0.3, 0.4, 0.5, 0.6$.

c) The ratio of non-medical expenditure and medical expenditure C_h/H. According to National Bureau of Statistics (2017), in 2016, the total medical expenditure in China is 4634.49 billion CNY; the total expenditure is 29344.3 billion CNY; and the out-of-pocket medical expenditure is 1333.8 billion CNY. Thus, we have $C_h/H \approx 6.04$.

d) The coefficient of relative risk aversion γ. According to Wang and Cai (2011), we consider $\gamma = 3, 4, 5, 6$.

Then, we simulate the optimal reimbursement ratio of medical insurance for Sample 50, shown in Table S.2.

Table S.2: Optimal reimbursement ratio of medical insurance for Sample 50 (Unit: %)

	$\varepsilon_{H,b} = 0.3$		$\varepsilon_{H,b} = 0.4$	
	$\varphi_1 = 0$	$\varphi_1 = 11.7\%$	$\varphi_1 = 0$	$\varphi_1 = 11.7\%$
$\gamma = 3$	49.4	70.2	35.9	56.2
$\gamma = 4$	61.7	77.5	51.3	66.8
$\gamma = 5$	69.1	81.9	60.7	73.3
$\gamma = 6$	74.2	84.9	67.1	77.7
	$\varepsilon_{H,b} = 0.5$		$\varepsilon_{H,b} = 0.6$	
	$\varphi_1 = 0$	$\varphi_1 = 11.7\%$	$\varphi_1 = 0$	$\varphi_1 = 11.7\%$
$\gamma = 3$	23.6	43.5	12.4	31.8
$\gamma = 4$	41.8	57.1	33.0	48.1
$\gamma = 5$	53.0	65.4	45.8	58.1
$\gamma = 6$	60.5	71.0	54.5	64.9

According to Table S.2, for any $\{\gamma, \varepsilon_{H,b}\}$ combination, the optimal reimbursement ratio of medical insurance is always smaller when we do not consider the effect of health on the marginal utility of consumption than when we consider the effect. For example, when $\gamma = 4$ and $\varepsilon_{H,b} =$

0.5, if we do not consider such effect, the optimal reimbursement ratio is 41.8%. However, if we consider the effect of health, the optimal reimbursement ratio is 57.1%. This example shows that if we do not consider the effect of health on the marginal utility of consumption, the optimal reimbursement ratio of medical insurance may be underestimated by 15.3%.

S.3.2. Optimal saving rate

Consider a two-period model with optimal saving. An individual earns wage w in the first period and save some amount s for the consumption in the second period. Considering the value of time, each unit of saving can generate e^{rT} unit of consumption in the second period, in which r is the real return and T is the length between two periods. Individuals are healthy in the first period, while they have probability p to be sick. When we consider the possible effect of health on the marginal utility of consumption, we assume that the marginal utility of consumption in the sick state is $u_s'(C) = (1+\varphi_1)u_h'(C) = (1+\varphi_1)u'(C)$. Suppose a CRRA utility function $C^{1-\gamma}/(1-\gamma)$. Then, the intertemporal utility maximization problem is:

$$\max_s u_h(C_1) + e^{-\delta T}[(1-p)u_h(C_2) + pu_s(C_2)] \tag{S.13}$$

$$s.t. s = w - C_1$$

$$C_2 = e^{rT}s.$$

The solution of this problem gives the optimal saving:

$$s^* = \frac{w}{1+[1-p+p(1+\varphi_1)]^{\frac{-1}{\gamma}} e^{rT+\frac{(\delta-r)T}{\gamma}}}, \tag{S.14}$$

and accordingly the optimal saving rate:

$$\frac{s^*}{w} = \frac{1}{1+[1-p+p(1+\varphi_1)]^{\frac{-1}{\gamma}} e^{rT+\frac{(\delta-r)T}{\gamma}}}. \tag{S.15}$$

The simulation for the value of s^*/w needs the following parameter values.

a) The effect of health status on the marginal utility of consumption φ_1. We use the estimation results for Sample 50 in the baseline regression ($\varphi_1 = \beta_1/\beta_4 = 11.7\%$).
b) The coefficient of relative risk aversion γ. According to Wang and Cai (2011), we consider $\gamma = 3, 4, 5, 6$.
c) The probability of being sick in the second period p. According to the descriptive statistics, we use $p = 0.67$.
d) The real return of savings r. A realistic value of r can be 1%.
e) The length between two periods T. We use $T = 25$.
f) The discount rate δ. Realistic values of δ can be 4%, 5%, 6%, and 7%.

Then, we simulate the optimal saving rate for Sample 50, shown in Table S.3.

According to Table S.3, for any $\{\gamma,\ \delta\}$ combination, the optimal saving rate is always smaller when we do not consider the effect of health on the marginal utility of consumption than when we consider the effect. For example, when $\gamma = 4$ and $= 4\%$, if we do not consider such effect, the optimal saving rate is 39.2%. However, if we consider the effect of health, the optimal saving rate is 39.7%. This example shows that if we do not consider the effect of health on the marginal utility of consumption, the optimal saving rate may be underestimated by 0.5%.

To summarize, according to our simulation results, there might exist bias in the current medical reimbursement policy. It might be helpful for policy makers to consider the effect of health status on the marginal utility of consumption. To some extent, an increase in the reimbursement ratio of

medical insurance will improve social welfare. In addition, there might also exist the possibility of underestimation of optimal saving rate, which is not conducive to the scientific evaluation of saving, investment and economic growth. Therefore, considering reasonably the effect of health status may benefit the economic development of China, especially under the background of population ageing.

Table S.3: Optimal saving rate for Sample 50 (Unit: %)

	$\delta = 4\%$		$\delta = 5\%$	
	$\varphi_1 = 0$	$\varphi_1 = 11.7\%$	$\varphi_1 = 0$	$\varphi_1 = 11.7\%$
$\gamma = 3$	37.8	38.3	35.8	36.4
$\gamma = 4$	39.2	39.7	37.8	38.2
$\gamma = 5$	40.1	40.5	38.9	39.3
$\gamma = 6$	40.7	41.0	39.7	40.0
	$\delta = 6$		$\delta = 7$	
	$\varphi_1 = 0$	$\varphi_1 = 11.7$	$\varphi_1 = 0$	$\varphi_1 = 11.7$
$\gamma = 3$	33.9	34.5	32.1	32.6
$\gamma = 4$	36.3	36.7	34.9	35.3
$\gamma = 5$	37.8	38.1	36.6	36.9
$\gamma = 6$	38.7	39.0	37.8	38.1

Reference

1. Finkelstein, A.; Luttmer, E.F.P.; Notowidigdo, M.J. What good is wealth without health? The effect of health on the marginal utility of consumption. *NBER Working Paper No. 14089* **2008.** URL: https://www.nber.org/papers/w14089
2. Chetty, R. A new method of estimating risk aversion. *American Economic Review* **2006**, *96*, 1821-1834. doi:10.1257/aer.96.5.1821.
3. Bao, Z.; Zhao, Y. A study on the optimal payment level of rural residents' basic medical insurance. *Insurance Studies* **2017**, *10*, 102-117. (in Chinese) doi:10.13497/j.cnki.is.2017.10.009
4. Wang, S.; Cai, M. Measurement and determinants of coefficients of risk aversion of Chinese residents: An empirical study based on investment behavior. *Journal of Financial Research* **2011**, *8*, 192-206. (in Chinese)
